# Correspondence of functional connectivity gradients across human isocortex, cerebellum, and hippocampus

**DOI:** 10.1038/s42003-023-04796-0

**Published:** 2023-04-12

**Authors:** Yuta Katsumi, Jiahe Zhang, Danlei Chen, Nada Kamona, Jamie G. Bunce, J. Benjamin Hutchinson, Mathew Yarossi, Eugene Tunik, Bradford C. Dickerson, Karen S. Quigley, Lisa Feldman Barrett

**Affiliations:** 1 Department of Neurology, Massachusetts General Hospital and Harvard Medical School, Boston, MA 02114 USA; 2 Department of Psychology, Northeastern University, Boston, MA 02115 USA; 3 Department of Biology, Northeastern University, Boston, MA 02115 USA; 4 Department of Psychology, University of Oregon, Eugene, OR 97403 USA; 5 Department of Electrical and Computer Engineering, Northeastern University, Boston, MA 02115 USA; 6 Department of Physical Therapy, Movement, and Rehabilitation Science, Northeastern University, Boston, MA 02115 USA; 7 Department of Psychiatry, Massachusetts General Hospital and Harvard Medical School, Boston, MA 02114 USA; 8 Athinoula A. Martinos Center for Biomedical Imaging, Massachusetts General Hospital and Harvard Medical School, Boston, MA 02114 USA

**Keywords:** Cognitive neuroscience, Human behaviour

## Abstract

Gradient mapping is an important technique to summarize high dimensional biological features as low dimensional manifold representations in exploring brain structure-function relationships at various levels of the cerebral cortex. While recent studies have characterized the major gradients of functional connectivity in several brain structures using this technique, very few have systematically examined the correspondence of such gradients across structures under a common systems-level framework. Using resting-state functional magnetic resonance imaging, here we show that the organizing principles of the isocortex, and those of the cerebellum and hippocampus in relation to the isocortex, can be described using two common functional gradients. We suggest that the similarity in functional connectivity gradients across these structures can be meaningfully interpreted within a common computational framework based on the principles of predictive processing. The present results, and the specific hypotheses that they suggest, represent an important step toward an integrative account of brain function.

## Abstract (web summary)

Analysis of functional MRI data from the Human Connectome Project and Brain Genomics Superstruct Project reveals common functional gradients among the human isocortex, cerebellum, and hippocampus.

## Introduction

Research capitalizing on genetic, histological, and neuroimaging data has started to reveal brain structure-function relationships at various levels of analysis within the cerebral cortex^1–16^. A key technique in elucidating such relationships involves mapping of high dimensional biological features to lower-dimensional manifold representations, also known as gradients^17–19^. Gradient mapping reduces the dimensionality of complex data through dimensional decomposition techniques (e.g., diffusion map embedding, principal component analysis). When applied to brain connectivity data, the resulting gradients can be interpreted as low-dimensional spatial representations of continuous transitions in connectivity profiles within or across brain structures.

Prior work computing connectivity gradients in humans has most often used blood-oxygen-level-dependent (BOLD) functional magnetic resonance imaging (fMRI) signal collected when a person is not being deliberately probed with an external task—so-called resting state or intrinsic activity^20,21^. Previous studies using various analytical approaches have consistently identified a few functional connectivity gradients within the isocortex^8,22–25^. One gradient is referred to as the association-sensorimotor gradient (also variably called a transmodal-unimodal gradient^23^) that describes gradual changes in the similarity of connectivity profiles from heteromodal regions typically considered part of the default mode network^6,26–28^ to the primary sensory areas in the cerebral cortex. This gradient has been recently characterized as a domain-general axis along which several biological features are organized, including cortical thickness, cerebral metabolism, intracortical myelination, neuronal size and density, evolutionary cortical expansion, embryonic development, and allometric scaling^29,30^. We refer to another connectivity gradient that is consistently identified in the literature as the representation-modulation gradient (also variably called a multiple demand gradient^7,31^, which is anchored at one end by regions part of the default mode, somatosensory/motor, and visual functional networks and at the other end by regions part of the attentional (i.e., salience, frontoparietal, and dorsal attention) networks. This gradient distinguishes isocortical ensembles involved in the representation of higher-dimensional sensory signals (in somatosensory and visual areas) or their compressed, lower-dimensional multimodal summaries (in the default mode network)^32^ from those ensembles that are thought to modulate these representations via processes such as attention regulation, goal maintenance, and strategy selection^33–36^. Another gradient that is commonly reported in the literature on functional connectivity gradients distinguishes between the visual network and the somatosensory/motor network^22,23,37^. It is not immediately clear what this gradient represents: It may suggest a segregation of exteroceptive sensory systems, but it may also suggest a distinction in the properties of sensory signals as they arise from different sensory surfaces (e.g., signal frequency, degree of compression when entering the isocortex, or even proximity to visceromotor control)^25^.

Beyond the isocortex, research has also identified connectivity gradients that characterize the functional organization of the cerebellum^38,39^ and the hippocampus^40–42^ in terms of their intrinsic connectivity within themselves or in relation to the isocortex. However, the degree of correspondence between these connectivity gradients, and their similarity to intrinsic connectivity gradients within the isocortex, has not been well characterized. The functional similarity of these gradients is suggested by numerous studies describing coordinated learning systems across the isocortex and cerebellum^43,44^, isocortex and hippocampus^45,46^, and cerebellum and hippocampus^47–51^. Despite this evidence, to our knowledge, no published study to date has systematically examined how the functional organization of the isocortex, the cerebellum, and the hippocampus relate to one another in terms of macroscale connectivity gradients defined based on BOLD fMRI data.

In the present study, we investigated the correspondence between intrinsic functional connectivity gradients of the isocortex, and of the cerebellum and the hippocampus in relation to the isocortex, using BOLD fMRI data collected at wakeful rest from healthy young adult participants in the Human Connectome Project^52^ (HCP, *n* = 1003) as our primary sample, and in participants in the Brain Genomics Superstruct Project^53,54^ (GSP, *n* = 1102) as our validation sample. In addition to group-level replication, we also assessed the degree to which the correspondence in functional connectivity gradients across structures was replicable at the level of individual participants (see Supplementary Information). Following prior published work on functional connectivity gradients, we derived functional connectivity gradients for each structure via diffusion map embedding, an established technique to nonlinearly reduce the dimensionality of large-scale connectivity data^19,55^. We chose to analyze these data from the perspective of the isocortex to consider how gradients of cerebellar and hippocampal connectivity to the isocortex would align with connectivity gradients within the isocortex, focusing on the three most dominant gradients in each structure (see Methods).

## Results

### Characterization of functional connectivity gradients in the cerebellum and the hippocampus in relation to the isocortex

To characterize the functional organization of the cerebellum and the hippocampus in relation to the isocortex, we first constructed group average functional connectivity (i.e., Fisher *Z*-transformed Pearson’s correlation coefficient) matrices between all cerebellar voxels and all isocortical surface vertices, as well as between all hippocampal voxels and all isocortical vertices, which were used as inputs for diffusion map embedding. The resulting connectivity gradients in this study, therefore, represented how similar a given pair of voxels are within each non-isocortical structure in terms of their patterns of connectivity with the isocortex. This approach is consistent with that of published work on the hippocampus and subcortical structures, in which functional connectivity gradients were defined based on the pattern of connectivity between these structures and the isocortex^7,39,41,42,56,57^.

**Fig. 1:**
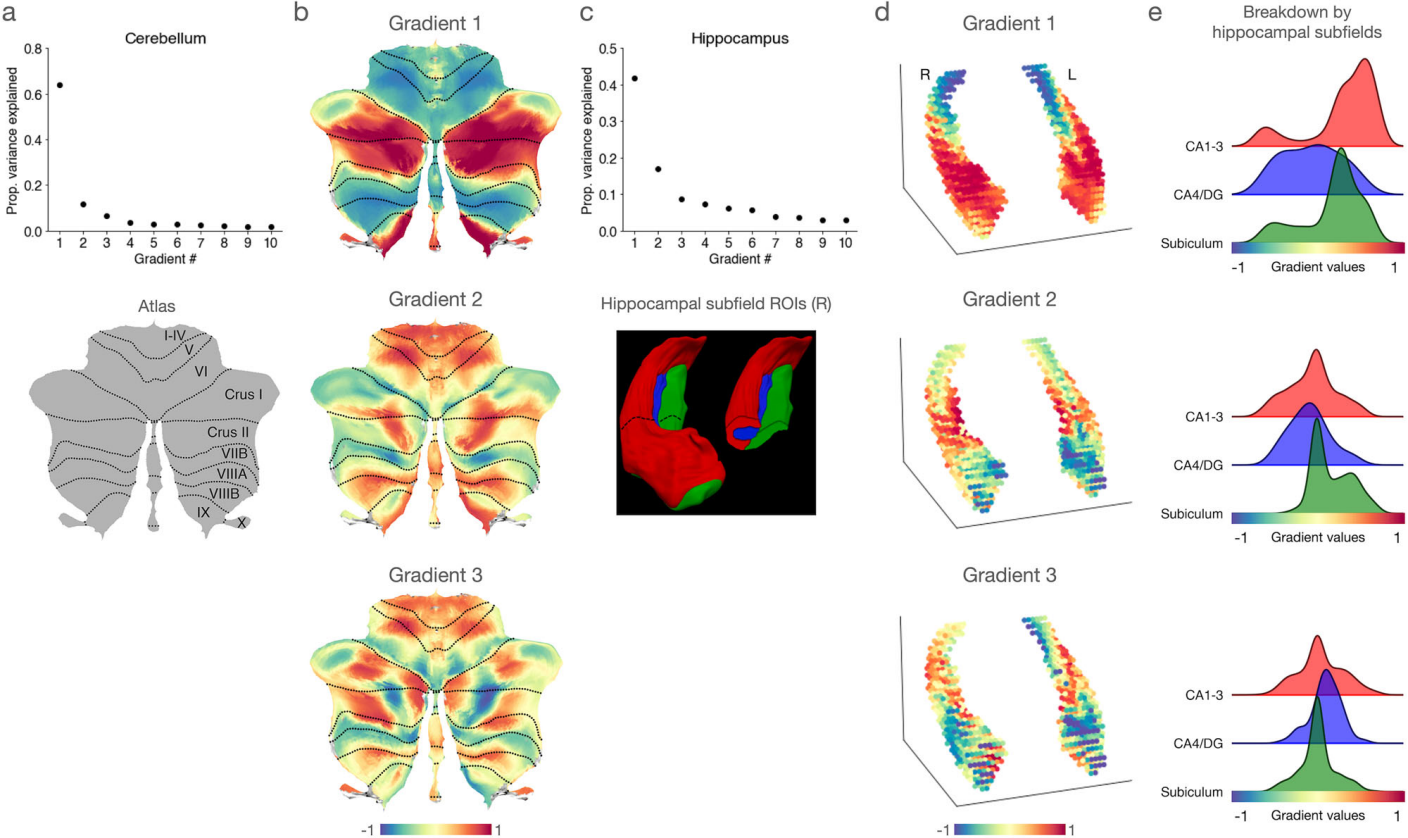
Functional connectivity gradients of the cerebellum and the hippocampus in relation to the isocortex in humans (*n* = 1003). **a** The scree plot illustrates the proportion of variance explained by each of the ten functional connectivity gradients derived from intrinsic connectivity between the cerebellum and the isocortex. The flatmap as a reference of the cerebellar lobules was reproduced from ref. ^38^ with permission. **b** The three most dominant gradients of cerebellar-isocortical connectivity**. c** The scree plot illustrates the proportion of variance explained by each of the ten functional connectivity gradients derived from the intrinsic connectivity between the isocortex and the hippocampus (top). A figure illustrating the subfields in the right hippocampus (red = CA1–3, blue = CA4-DG, green = subiculum) was reproduced from ref. ^58^ with permission. **d** The three most dominant gradients of the hippocampal-isocortical connectivity. **e** The ridge plot depicts the distribution of gradient values per hippocampal subfield group.

For intrinsic cerebellar-isocortical connectivity, the top three gradients collectively explained >80% of the variance in the data, with Gradient 1 accounting for >60%, Gradient 2 accounting for >10%, and Gradient 3 accounting for >5% (Fig. 1a). Gradient 1 captured a bilateral dissociation of lobules I-IV, V, and VI and lobule VIII from the posterior part of Crus I and II and the medial part of lobule IX, whereas Gradient 2 distinguished bilaterally the anterior parts of Crus I and Crus II along with lobule VIIB from the rest of the cerebellar cortex. Gradient 3 revealed a more complex dissociation that does not clearly follow the anatomical boundaries of cerebellar lobules, which was anchored at one end by lobules I-IV, V, the posterior-most and mid portions of Crus I and II, lobules VIIIB, IX, and X (Fig. 1b).

For intrinsic hippocampal-isocortical connectivity, the top three gradients together explained >65% of the variance in the data within each hemisphere, with Gradient 1 accounting for >40%, Gradient 2 accounting for >15%, and Gradient 3 accounting for >8% of the variance in the data (Fig. 1c). Gradient 1 generally captured spatial variation in functional connectivity along the longitudinal axis of the hippocampus, whereas the variation captured by Gradient 2 was observed in both the longitudinal axis and the medial-lateral axis. Gradient 3 revealed a more nuanced mixture of these axes and was anchored at one end by the postero-lateral subregions and at the other by the antero-medial subregions (Fig. 1d). To understand these gradients in terms of hippocampal microstructure, we performed Mann–Whitney tests (two-sided) to compare the distribution of gradient values for the major hippocampal subfields: Subicular complex, CA1–3, and CA4-dentate gyrus (CA4-DG), which were derived from the established segmentation protocol^58^ (Fig. 1e). This analysis revealed that Gradient 1 distinguished between the CA subfields, with CA1–3 showing the highest values overall, followed by subiculum and then the CA4-DG (*p* ≤ 0.001 for all pairwise comparisons). Gradient 2 was anchored by the subiculum at one end and CA1–3 and CA4-DG at the other (*p* < 0.001), with no significant differences between CA1–3 and CA4-DG (*p* ≤ 0.16). Gradient 3 likewise distinguished between the three hippocampal subfield groups, with the CA4-DG showing higher values than CA1–3 (*p* ≤ 0.004) and the subiculum (*p* ≤ 0.001); CA1–3 also showed higher Gradient 3 values than the subiculum (*p* ≤ 0.02).

### Correspondence of functional connectivity gradients between the isocortex, cerebellum, and hippocampus

Having characterized the major functional connectivity gradients of the cerebellum and the hippocampus in relation to the isocortex, we next investigated the relationships between these gradients and functional connectivity gradients identified within the isocortex. To derive isocortical connectivity gradients, we constructed a subset of the whole-brain group average functional connectivity matrix with all isocortical vertices, which was used as input to diffusion map embedding. From this analysis, we identified the top three gradients that describe the maximal variance in explaining the organization of functional connectivity patterns within the isocortex. These isocortical gradients replicated those identified by previous studies, with Gradient 1 distinguishing the default mode and frontoparietal networks from the exteroceptive sensory (e.g., somatosensory, visual) as well as salience networks, Gradient 2 distinguishing the visual network from the somatosensory/motor network, and Gradient 3 distinguishing the default mode and exteroceptive sensory networks from the frontoparietal and salience networks^23,25^ (Supplementary Fig. 1).

**Fig. 2:**
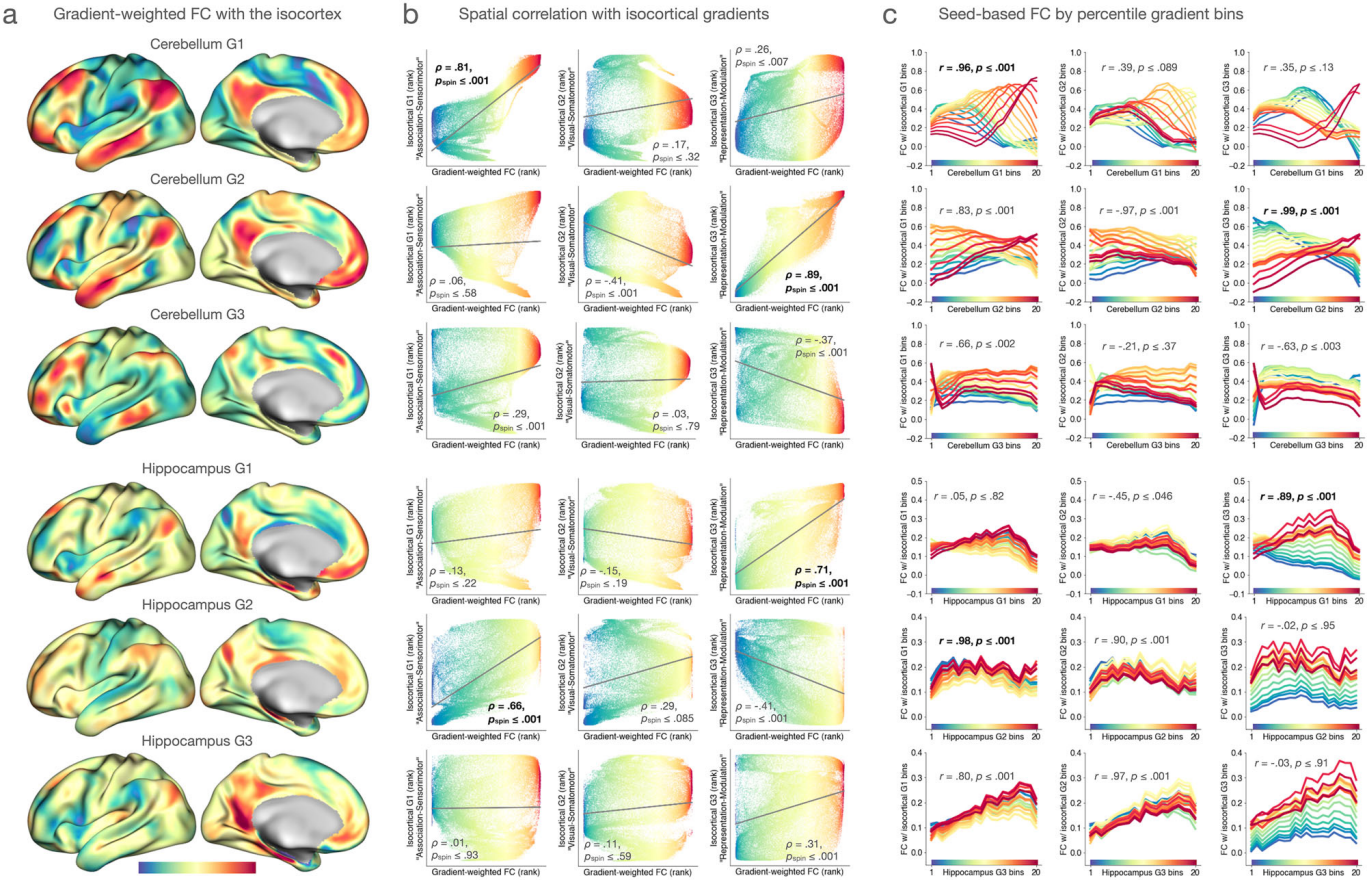
Correspondence between non-isocortical and isocortical functional connectivity gradients. **a** Gradient-weighted functional connectivity maps were calculated by multiplying voxel-wise isocortical functional connectivity maps of the cerebellum (or hippocampus) by cerebellar (or hippocampal) connectivity gradient values. The resulting surface maps thus characterize the topography of the isocortical functional connectivity variation that each non-isocortical gradient represents. **b** Spatial correspondence between each pair of a gradient-weighted connectivity map and an isocortical connectivity gradient was assessed by Spearman’s rank correlation; *p* values were computed while controlling for spatial autocorrelation via spin permutation tests^60^. **c** Based on the percentile ranks associated with voxel-wise gradient values, each non-isocortical and isocortical gradient was discretized into 20 spatially non-overlapping bins. These bins were used to compute functional connectivity between each unique pairwise combination of gradient bins across structures. For each non-isocortical gradient bin, Pearson’s correlation coefficient was calculated between isocortical gradient bin indices (1–20) and functional connectivity values associated with them, thus yielding a single correlation coefficient per non-isocortical bin. In the next step, Pearson’s correlation coefficient was calculated between non-isocortical gradient bin indices (1–20) and these isocortical correlation coefficients. This procedure, therefore, resulted in one correlation coefficient for each pair of non-isocortical and isocortical gradients, shown here in each panel, representing the extent to which these gradients correspond to one another in terms of functional connectivity. All *p* values are based on two-sided hypothesis testing. For each non-isocortical gradient, the strongest correlation is shown in bold font.

Next, we calculated intrinsic functional connectivity maps for each of these structures weighted by their voxel-wise gradient values, using the same procedure as published work^25,39,59^. For each cerebellar and hippocampal gradient, this procedure yielded an isocortical map characterizing the variability in isocortical functional connectivity along a given non-isocortical gradient (Fig. 2a). We then quantitatively assessed the degree of spatial correlation between these gradient-weighted functional connectivity maps and isocortical connectivity gradients by computing vertex-wise Spearman’s rank correlations, while controlling for spatial autocorrelations and statistical significance assessed via spin permutation tests^60^ (Fig. 2b). This analysis revealed that isocortical Gradient 1 showed the strongest spatial correspondence with the weighted connectivity maps of cerebellar Gradient 1 (*ρ* = 0.81, *p*_spin_ ≤ 0.001) and of hippocampal Gradient 2 (*ρ* = 0.66, *p*_spin_ ≤ 0.001) compared with the other gradient-weighted connectivity maps of these non-isocortical structures. In contrast, isocortical Gradient 3 showed the strongest correspondence with the weighted connectivity maps of cerebellar Gradient 2 (*ρ* = 0.89, *p*_spin_ ≤ 0.001) and of hippocampal Gradient 1 (*ρ* = 0.71, *p*_spin_ ≤ 0.001) relative to the other weighted connectivity maps of these structures.

To further clarify the relationships between functional connectivity gradients across structures, we additionally performed conventional seed-based analyses to estimate cerebello-isocortical and hippocampo-isocortical functional connectivity using discretized functional connectivity gradients of each structure as seed regions of interest. Here, each cerebellar, hippocampal, and isocortical functional connectivity gradient derived from the same group average connectome data was first split into 20 spatially-discontiguous bins based on the percentile ranks of vertex-/voxel-wise gradient values. We then calculated bin-to-bin functional connectivity for each pairwise combination of the three structures. Specifically, for each pair of non-isocortical and isocortical gradients, we calculated pairwise functional connectivity based on 20 non-isocortical bins and 20 isocortical bins and examined the magnitude of isocortical connectivity changes along a non-isocortical gradient. If there exists a one-to-one relationship in functional connectivity between a given pair of non-isocortical and isocortical gradients, we expect that the bins anchoring the corresponding ends of the same gradient would be maximally correlated with one another. To test this idea, we first quantified the magnitude of functional connectivity between each non-isocortical gradient bin and 20 isocortical gradient bins. We then computed a single Pearson’s correlation coefficient based on these 20 functional connectivity estimates and bin indices (1–20) along an isocortical gradient to represent the pattern of isocortical functional connectivity for each non-isocortical gradient bin. Once computed for each non-isocortical gradient bin, we calculated the correlation coefficient between the magnitude of isocortical functional connectivity and bin indices (1–20) along a non-isocortical gradient; this procedure yielded one Pearson’s correlation coefficient that quantifies the strength of the linear relationship between a given pair of non-isocortical and isocortical gradients based on functional connectivity. This analysis revealed results largely consistent with the analysis of gradient-weighted functional connectivity discussed above, demonstrating that differences in functional connectivity between positions along isocortical Gradient 1 are most strongly associated with differences in functional connectivity along cerebellar Gradient 1 and hippocampal Gradient 2, whereas connectivity differences along isocortical Gradient 3 are most strongly associated with connectivity differences along cerebellar Gradient 2 and hippocampal Gradient 1 (Fig. 2c).

**Fig. 3:**
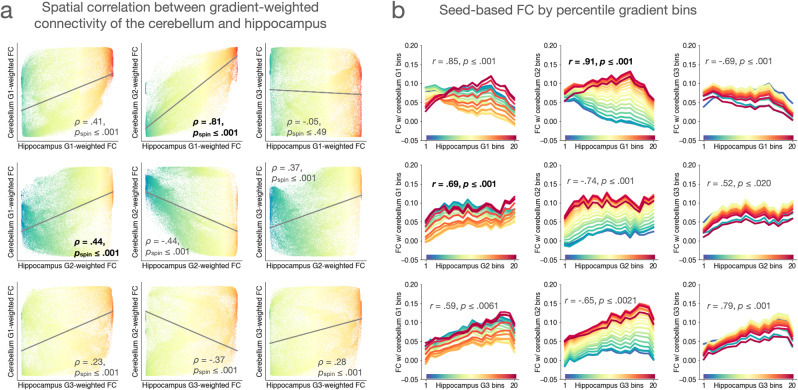
Correspondence between cerebellar and hippocampal functional connectivity gradients in relation to the isocortex. **a** Each panel represents the spatial association between gradient-weighted functional connectivity maps of the cerebellum and hippocampus (see Fig. 2a). **b** Results of seed-based functional connectivity analysis based on gradient bins in a given pair of cerebellar and hippocampal functional connectivity gradients in relation to the isocortex. All *p* values are based on two-sided hypothesis testing. For each non-isocortical gradient, the strongest correlation is shown in bold font.

Following the same analytical procedure, we additionally investigated the relationships between cerebellar and hippocampal connectivity gradients. Our hypothesis was that a pair of cerebellar and hippocampal gradients that correspond to the same isocortical gradient would also show the strongest correspondence with each other. Confirming this hypothesis, we found that cerebellar Gradient 2 and hippocampal Gradient 1, both of which corresponded most strongly to isocortical Gradient 3, showed a stronger spatial correlation in gradient-weighted functional connectivity maps as well as stronger linear relationship in seed-based functional connectivity with each other; a similar pattern of results was observed with cerebellar Gradient 1 and hippocampal Gradient 2, which corresponded most strongly to isocortical Gradient 1 (Fig. 3).

### Group-level replicability of the correspondence in functional connectivity gradients

To validate the pattern of results obtained within the discovery sample, we performed the same set of analyses using an independent sample of healthy young adults (*n* = 1102). Overall, the topography of functional connectivity gradients of the isocortex, the cerebellum, and the hippocampus was similarly observed across samples, although some differences in the distribution of gradient values were noted in higher-order gradients (Supplementary Figs. 2, 3). As observed in the discovery sample, hippocampal Gradient 1 primarily distinguished between the CA subfields, with CA1–3 showing higher gradient values compared with both the subiculum and CA4-DG (*p*’s ≤ 0.001). Hippocampal Gradient 2 also distinguished the subiculum from CA1–3 (*p* ≤ 0.001), whereas the difference between the subiculum and CA4-DG was not observed (*p* ≤ 0.84). Importantly, the correspondence in functional connectivity gradients across structures as examined by gradient-weighted functional connectivity maps and conventional seed-based analyses of functional connectivity via gradient bins both demonstrated the pattern of results consistent with those obtained with the discovery sample above (Supplementary Figs. 4, 5). These results strongly suggest that the observed results are robust to variations in data acquisition parameters and preprocessing methods.

### Single-participant replicability of correspondence in functional connectivity gradients

Finally, we assessed the degree to which the correspondence in functional connectivity gradients observed using group average data was replicable at the level of individual participants. To this end, we performed seed-based functional connectivity analysis as described above using group-defined gradient bins (Fig. 2c) but separately for each individual participant in the discovery sample. As a measure of single-participant replicability, we computed the percentage of participants whose pattern of seed-based functional connectivity was concordant with the corresponding group-level analysis. For instance, regarding cerebellar Gradient 1 and its association with isocortical gradients (Fig. 2c, top row), each participant had to exhibit maximal correlation in functional connectivity between cerebellar Gradient 1 and isocortical Gradient 1 compared with the other isocortical gradients for this participant to be identified as showing the pattern consistent with the group-level effect. This analysis revealed that the pattern of correspondence in functional connectivity gradients at the group level was overall replicable in the majority of our individual participants, although the extent of single-participant replicability varied across pairs of structures (Supplementary Table 1).

## Discussion

Accumulating evidence suggests that the organization of the isocortex^8,22,23,25,61–65^, the cerebellum^38,39^, and the hippocampus^7,40–42,66^ can be described with multiple gradients of functional features that map onto structural features in the human brain. In the present study, analyses of two large datasets (*N* > 2000) replicated two gradients identified by published research describing cerebellar and hippocampal functional connectivity to the isocortex^38,42^, and additionally revealed the topography of the third gradient in each structure that, to our knowledge, has not previously reported in the literature. We first discuss the results in terms of prior published evidence that characterizes the functional organization of one (non-isocortical) brain structure in terms of its connectivity profiles with the isocortex^7,39,42,57,67^, and then suggest that the corresponding gradients of functional connectivity might be understood as dominant functional organizing principles within a common computational framework. These results, and the specific hypotheses that they suggest, represent an important opportunity to build an integrative account of brain function.

Isocortical Gradient 1 (association-sensorimotor) primarily distinguishes between heteromodal regions part of the default mode network and the primary sensory cortical areas. Cerebellar Gradient 1, which showed strong correspondence to this isocortical gradient, has been similarly characterized as a gradual transition from areas involved in non-motor (i.e., cognitive, social, and emotional tasks) to those implicated in motor function^38^. When the organization of intrinsic functional network connectivity is considered^38,68^, cerebellar Gradient 1 is anchored at one end by the default mode and frontoparietal control networks and at the other end by the somatosensory/motor and salience networks, in a manner that is strongly similar to isocortical Gradient 1. Hippocampal Gradient 2, which also showed the strongest correspondence to this isocortical gradient, revealed a dissociation along the medial-lateral axis that differentiated the subiculum from the CA subfields. This result is in line with prior evidence characterizing distinct hippocampal subfields with variation in connectivity, computational roles, and myeloarchitectural maturation^69–72^. It has been shown that the subiculum exhibits stronger functional connectivity with the default mode network than the other subfields, whereas CA1–3 shows stronger connectivity with the somatosensory/motor and visual networks^42^, consistent with the observed correspondence in connectivity gradients.

Isocortical Gradient 3 (representation-modulation) distinguishes cortical areas as part of the default mode and primary sensory areas from functional networks such as the frontoparietal, salience, and dorsal attention networks. Cerebellar Gradient 2, which showed strong correspondence to this isocortical gradient, has been similarly interpreted as reflecting differences in attentional modulation, specifically in relation to task-focus^38^ that is required during tasks involving higher cognitive load^73^. This pattern is also consistent with prior work descriptively demonstrating that cerebellar Gradient 2 is associated with preferential connectivity with the default mode and somatosensory/motor networks at one end and the frontoparietal and salience networks at the other^38,68^. Hippocampal Gradient 1, which also showed the strongest correspondence to this isocortical gradient, has been described as capturing variation in functional connectivity along its longitudinal axis^40–42^, consistent with evidence identifying gradual changes in anatomical connectivity, gene expression, and electrophysiological response properties along this axis^66,74,75^. Prior work has identified the largest difference in intrinsic functional connectivity between the anterior and posterior subregions with respect to the frontoparietal and salience networks, with the posterior hippocampus showing stronger positive connectivity with these networks, and the default mode and somatosensory/motor networks at the anterior end^42^.

Isocortical Gradient 2, cerebellar Gradient 3, and hippocampal Gradient 3 require future research to clarify their possible functions. Cerebellar Gradient 3 appears to be anchored at one end by the frontoparietal and somatosensory/motor networks and at the other end by the default mode and salience networks. The default mode and salience networks are thought to constitute an intrinsic brain system for allostasis (i.e., predictive regulation of an animal’s body and its needs)^59,76,77^. It is, therefore, possible that this third gradient represents an axis of skeletomotor vs. visceromotor signals in the cerebellum, although these two signals are likely not independent of each other^78–80^. Hippocampal Gradient 3, which revealed a more complex topography along both the longitudinal and medial-lateral axes, also distinguishes between two of the hippocampal subfield groups. However, slight differences in the topography of this gradient were noted between the discovery and validation samples, suggesting that it may be sensitive to methodological variability. The two datasets examined in the current study, although similar in size, varied in numerous parameters associated with fMRI data acquisition (e.g., multi-band vs. single-band slice acquisition, spatiotemporal resolution of acquired images, number of runs), which may interact to influence the characteristics of BOLD signal and derivatives. In the context of overall similar results, prior studies of functional connectivity parcellation have also noted distinct topographical variability between the HCP and GSP datasets^81^. More research is needed to fully characterize hippocampal gradients; the use of high-field (e.g., 7 T) functional imaging would be especially useful in clarifying the microstructural properties of the hippocampus that each hippocampal connectivity gradient may be related to.

Importantly, the correspondence of functional connectivity gradients across these structures was largely replicable at the level of single participants, although the extent of this replicability also varied across structure pairs and as a function of the gradient order. To our knowledge, the current study is among the first to examine individual differences in the degree of correspondence in connectivity gradients. However, several studies have identified individual variation in the organization of functional connectivity gradients across participants and that this variation was predictive of various aspects of cognition in both healthy and clinical populations^22,37,56,82^. Extending this line of work, future studies might investigate how variation in gradient correspondence relates to individual differences in cognition and behavior.

The evidence of gradient correspondence may have important implications for clarifying the computational mechanisms of functional coordination among the isocortex, the cerebellum, and the hippocampus. In our recent work, we proposed that these two large-scale functional gradients can be interpreted as being an integral part of the intrinsic neural architecture supporting the implementation of predictive processing in the brain, including allostasis^25,59^. Below, we sketch this interpretation of the observed correspondence in functional connectivity gradients across structures with links to a clear theoretical formulation of predictive processing as an overarching computational framework. It is important to acknowledge upfront that empirical findings reported herein were based on interregional similarity in low-frequency fluctuations of BOLD signal at rest. By definition, the observed pattern of correspondence in functional connectivity gradients by itself cannot be taken as direct evidence for the flow of signals within a structure or across structures. Prior work has shown, for example, that the topography of brain states captured by hidden Markov modeling of resting fMRI data resembles different ends of functional connectivity gradients, suggesting that these gradients may be driven in part by the states occurring at rest^83^. These states are associated with structural architecture^84^, so this finding is not inconsistent with our hypothesis. Moreover, it was precisely because of the structural and other biological features that characterize isocortical Gradient 1 that we originally hypothesized its role in predictive processing^76,77,85–87^. The evidence of coordinated gradients builds on these original ideas to suggest hypotheses about the domain-general computations that may be associated with functional connectivity gradients. Future research needs to directly test this computational hypothesis regarding information flow using techniques that allow for causal inference.

Predictive processing is emerging as a powerful neurocomputational hypothesis that accounts for diverse psychological and biological functions subserved by a brain^87–90^. To date, predictive processing hypotheses have been offered to describe the computational capacities of several structures within the vertebrate brain, including the cerebral cortex in general^76,77,85,86,88,91–93^, the hippocampus^94–97^, and the cerebellum^43,98–100^. A variety of specific computational proposals abound, but they are generally united by three components that are thought to be implemented in a hierarchical arrangement in the brain’s architecture: (1) prediction signals that the brain generatively constructs using memory—or alternatively, an internal model^101^; (2) prediction errors that learn the differences between signals carrying sensory information predicted by the brain and sense data originating from the sensory surfaces of the body; and (3) precision signals that adjust the strength and durability of (1) and (2)^102,103^. Prediction errors are potential teaching signals, but their capacity to update predictions is thought to depend on how they are weighted by precision signals, which are interpreted as the predicted value of the allostatic information they will provide, or salience^76,104^. Prediction signals are also thought to be weighted by their estimated value to explain the incoming sense data, which may correspond to their estimated prior probabilities^76,102,103^. Predictive processing reduces uncertainty as an animal moves around in an ever-changing but only partly predictable world. Learning accumulates as an internal model of the animal’s body in the world by which the brain’s top-down predictions emerge and implement allostasis and from which movements and perceptions arise, all of which can be achieved in a metabolically efficient manner^59^.

It is hypothesized that the cytoarchitectural properties of the isocortex support the flow of prediction and prediction error signals (also referred to as feedback and feedforward signals^1,2,105^ as discussed in refs. ^59,76,87^) based on laminar development: Prediction signals are hypothesized to flow from deep layers of less granular areas (e.g., agranular, limbic cortices without layer IV) to the superficial layers of dysgranular cortices (e.g., where layers II and III are differentiated and layer IV is rudimentary); from the deep layers of dysgranular cortices to the superficial layers of granular (six-layered) cortices (e.g., eulaminate cortices with well-defined layer IV and upper layers); and from the deep layers of eulaminate cortices to the superficial layers of koniocortex (with the most well-developed layer IV and upper layers, e.g., primary visual cortex). Conversely, prediction error signals are hypothesized to flow from superficial layers of more granular cortices to deep layers of less granular cortices. As prediction error signals flow from sensory regions (whose upper layers contain many smaller pyramidal neurons with fewer connections) to limbic and other heteromodal regions in the frontal cortex (whose upper layers contain fewer but larger pyramidal neurons with many more connections), it is compressed and reduced in dimensionality^30^. A growing number of experimental studies have also verified signal flow or behavioral effects that are consistent with these hypotheses - e.g., evidence from electrophysiological recordings suggests prediction signal ensembles oscillate synchronously in the alpha and beta frequency bands, whereas prediction error signals oscillate in the higher-frequency gamma range^92,106,107^.

The cytoarchitectural gradient described above is overall topographically consistent with the isocortical association-sensorimotor functional gradient, suggesting its role in supporting the flow of prediction and prediction error signals. At the association end, the regions within the default mode network are thought to construct highly compressed multimodal representations, which enable the initiation of prediction signals guiding motor actions and making perception possible^76,90,108,109^. The topographical features of the default mode network may enable these computations, as cortical regions part of this network are maximally divergent from systems including primary exteroceptive sensory areas^23,110^. The frontoparietal network is hypothesized to play a major role in (1) estimating the precision of prediction signals by suppressing those with weaker priors and (2) creating and maintaining prediction signals longer than it takes to process incoming prediction errors, when necessary^76,90^. This hypothesis is consistent with available evidence showing that one of the frontoparietal network’s subnetworks acts as an extension of the default mode network to regulate complex introspective processes^111^. It is also in line with current evidence that these two networks exhibit longer timescales of cortical processing than the other cortical networks^64,112^.

At the sensorimotor end, exteroceptive sensory networks process signals that correspond to low-level sensory predictions that have been decompressed (as probabilistic inferences) from the compressed multimodal summaries in the association regions. We hypothesize that all cortical neurons process signals that continually confirm or refine the prediction signals they receive (i.e., prediction errors)^113^. The prediction errors at the sensorimotor end are closer in dimensionality to the sensory signals in the periphery, while the prediction errors at the association end constitute the compressed multimodal summaries of those lower-level signals.

The salience network is thought to estimate the precision of prediction errors by tuning the gain on these signals as they propagate from the body’s sensory surfaces, representing confidence in the reliability and quality of incoming sense data and their predicted relevance for allostasis. It is, therefore, possible that this network helps the brain adjust its internal model to the body’s energetic conditions^76^. This hypothesis is in line with other views of salience network function that emphasize its role in both attention regulation^36,114–116^ and multisensory integration^27,117^. The representation-modulation gradient in the isocortex, then, might be interpreted as an axis of the functional organization that differentiates ensembles associated with the representation of prediction and prediction error signals (i.e., anchored in nodes of the default mode and sensory networks) from those involved in the implementation of attentional modulation to set the precision of these signals (with nodes from the frontoparietal and salience networks).

There are also accounts of brain structure-function correspondence that are complementary to predictive processing hypotheses and consistent with the current findings. For example, research in comparative neuroscience has linked evolutionary changes in general brain-scaling functions^118^ to expanded association cortices in humans when compared with other primates, including other great apes^119,120^. This expansion potentially allows for increased information compression and dimensionality reduction, suggesting the possibility that human brains are capable of multimodal summaries characterized by greater abstraction^76,121^. This perspective has been integrated into the predictive processing account^59,76^, but requires a further empirical test.

The observed correspondence in functional connectivity gradients across the isocortex, cerebellum, and hippocampus can be used as a starting point for developing a unified, integrative view of brain function, building upon prior work investigating the computational capacities of these structures based on the principles of predictive processing. Although speculative, we hypothesize that the isocortex, cerebellum, and hippocampus might integrate over the same signals, but emphasize and/or construct different features to modulate one another as they construct prediction, prediction error, and precision signals in the service of allostasis, owing to their extensive connectivity with one another and with the subcortical structures implicated in the processing of these signals^59^.

It has been traditionally thought the cerebellum estimates the sensory state of the body by predicting the consequences of motor commands^43,44,100^, possibly as a means to compensate for delays in sensory feedback^122,123^. In visual perception, the cerebellum is thought to be critical for predicting incoming sensory information based on sequence detection and updating predictions based on the statistics of the sensory environment^124–126^. Extending these accounts, we hypothesize that the cerebellum computes sensory prediction error signals (and perhaps predicts the sensory prediction errors within the isocortex) to adjust signals of various cortical ensembles faster than isocortical sensory prediction error signals can be computed^59^.

The cerebellum may also be involved in estimating the precision of isocortical sensory prediction errors. During motor learning, the brain controls error sensitivity (i.e., the extent to which the brain changes the motor commands in the trial following an error) by learning relatively more from small and consistent errors than from larger and variable ones^127,128^. This learning mechanism depends critically on the memory of errors that accumulate during training, which exists independently of two traditional forms of motor memory (memory of perturbations and of actions)^127^. Although motor learning can occur on different timescales with different error sensitivities^129^, the memory of errors is thought to exert its influence through the error sensitivity of the fast learning process^127^. Therefore, an additional possibility is that the cerebellum rapidly estimates the reliability of sensory prediction errors in the isocortex, conveying this information to parts of the isocortex (e.g., the premotor areas such as the anterior mid-cingulate cortex within the salience network) where it is further used to update precision estimates about sensory prediction error signals.

The hypothesis that the cerebellum can exert rapid modulation of signals in the isocortex via coordinated functional gradients is further supported by available microanatomical and connectivity evidence. The majority of cerebellar neurons are granule cells, which can generate action potentials that are relatively short-lived and at much higher frequencies than neurons in the cerebral cortex^122^. Deep cerebellar nuclei, which are the gateway of cerebellar output, can also be modulated to fire up to 100+ Hz on average^130^. Despite the fact that the cerebral cortex and the cerebellum are connected to each other only by way of polysynaptic projections^68,131^, numerous nonprimary sensorimotor (e.g., parietal association, parahippocampal, occipitotemporal, and prefrontal) areas of the cerebral cortex project to the cerebellar cortex via the cortico-ponto-cerebellar paths^132^. The isocortical areas that project to specific parts of the cerebellar cortex via the pons are also the target of efferent projections from the same cerebellar cortical areas via the thalamus^133–135^. These parallel, reciprocally-connected cerebello-isocortical circuits might provide an anatomical substrate for the correspondence in functional connectivity gradients between the isocortex and the cerebellum identified in this study, further supporting a domain-general view of cerebello-isocortical interaction. The cerebellum also exhibits monosynaptic or polysynaptic connections with several subcortical structures that are known to play an important role in allostasis, including the hypothalamus, periaqueductal gray (PAG), nucleus of the solitary tract, and amygdala^136,137^, suggesting a critical contribution of this structure to predictive regulation of the body.

The hippocampus is thought to generate prediction signals^45,94,97,138,139^ and facilitate reweighting of signals in the isocortex^46^. Specifically, it may help ensure that the subsequent prediction signals generated by the internal model of the isocortex are not slaves to the statistics of the external sensory environment and instead are more in line with the animal’s goals (i.e., weighted for the current and predicted conditions of the body’s internal milieu)^46^. This mechanism likely draws upon the functional loop between the hippocampus, the entorhinal cortex, and the isocortex. Isocortical afferents to the hippocampus carry highly compressed, multimodal summaries of sensory information via the entorhinal cortex^140^, whose Layer II and III project widely to the DG, CA1-CA4, and the subiculum via the perforant path^75,141^. Subcortical projections to the hippocampus include those from the medial septum, amygdala, anterior thalamic nuclei, supramammillary nucleus of the hypothalamus, and brainstem nuclei such as the ventral tegmental area, PAG, and locus coeruleus^141,142^. By interfacing with isocortical ensembles at many levels of the predictive hierarchy, while receiving rich low-level sensory information from subcortical structures, the hippocampus, too, may intervene at multiple points of this hierarchy to modulate cortical signaling. This hypothesis is consistent with anatomical evidence identifying projections of CA1 and subiculum to Layer V and VI of the entorhinal cortex^140^ as well as widespread multimodal association areas in the isocortex, including the medial frontal cortex, temporal pole, orbitofrontal cortex, anterior and posterior cingulate cortices, parietal and inferotemporal cortices^143,144^ and to some extent lateral frontal cortex^143^.

This is warranted by the topographical organization of its connectivity gradients observed in the current study as well as the mapping of these gradients to isocortical functional networks^42^. Specifically, at the posterior end of the hippocampus (corresponding to the colder colored voxels of hippocampal Gradient 1; Fig. 1d), intrinsic functional connectivity was stronger with the isocortical attentional networks; in the middle and anterior portions of the hippocampus (corresponding to the warmer colored voxels of hippocampal Gradient 1 and Gradient 2), functional connectivity was stronger with the default mode network in the isocortex; in the anteriormost portion of the hippocampus (corresponding to the colder colored voxels of hippocampal Gradient 2), functional connectivity was stronger with the somatosensory/motor areas of the isocortex. The attentional-to-default and default-to-sensorimotor gradients in the hippocampus, therefore, may characterize the contribution of this structure to predictive processing in the brain, which involves the refinement of representations in the isocortex regardless of whether they are content-based or modulatory.

Coordinated gradients of connectivity suggest signal exchange between the cerebellum and the hippocampus, whose interaction is understudied thus far. Emerging evidence suggests the existence of a cerebello-hippocampal learning system^47–51,145,146^, although its computational and functional architecture are relatively less well studied when compared with the other learning systems discussed. Viral tracing studies have so far identified polysynaptic connections between these structures mediated by regions including the supramammillary nucleus of the hypothalamus, medial septum, and ventrolateral/laterodorsal thalamus^50,147^. There is also evidence pointing to the existence of direct connections between cerebellar and hippocampal subregions in humans^148^. The present findings reinforce the importance of testing specific hypotheses, for instance, about event segmentation and sequence processing in which both structures have been (separately) implicated^45,149–153^. Future work should investigate the complementary contributions of cerebello-isocortical, hippocampo-isocortical, and cerebello-hippocampal interactions to the brain’s internal model, which might be characterized by their dissociable involvement in processing different types of information and/or on different timescales.

The present results offer the opportunity to synthesize evidence across literatures into a common neurocomputational framework based on the principles of predictive processing. Our hypotheses, while speculative, illustrate the value of connectivity gradients in innovating specific questions about the computational aspects of brain function, with the association-sensorimotor and representation-modulation gradients as two common axes of information processing in the brain. Future work might specifically address these questions and probe modulation of connectivity gradient coordination across structures by explicit task demands or by clinical conditions in which neural mechanisms subserving predictive processing are hypothesized to be dysfunctional^154,155^. Future work might also consider expanding analysis space to the broader subcortex to more comprehensively take into account its functional organization^156,157^ and examine their contribution to predictive processing. If this framework bears fruit, it has the potential to offer a coherent, neurobiologically-inspired research program to unite the study of mind and behavior, collapsing the artificial boundaries between cognitive, perceptual, affective, motor, and even social phenomena. This evidence might also provide a common framework for understanding and treating the neurocomputational basis of mental disorders, neurodegenerative disorders, and physical disorders.

## Methods

### Datasets

We analyzed the fMRI data collected at wakeful rest from 1003 participants as part of the HCP WU-Minn Consortium^52^ (*M*_age_ = 28.71, SD_age_ = 3.71, 470 males, 533 females; four 15 min runs per participant) included in the HCP1200 2017 data release. All group-level analyses were conducted using the group average preprocessed whole-brain dense functional connectome (.dconn) data identifying temporal correlations between all cortical vertices and subcortical voxels. For individual-level analyses, we used preprocessed timeseries (.dtseries) data to derive estimates of functional connectivity, which were averaged across four runs. A full description of the data preprocessing pipelines implemented by the HCP is discussed elsewhere^158,159^. Briefly, each participant’s fMRI data underwent gradient distortion correction, EPI distortion correction, motion correction, spatial co-registration to structural reference, spatial normalization to template volumetric space, resampling to template surface space, volumetric and surface smoothing with a 2 mm Gaussian kernel, and were submitted to independent component analysis (ICA) for further artifact removal^160,161^. For inter-subject registration, feature-based alignment and the Multimodal Surface Matching Algorithm (MSMAII) were implemented^162,163^. Prior to the computation of the group average dense connectome, each dataset was temporally demeaned and had variance normalization applied^164^ and submitted to the group-level principal component analysis (PCA). The output of the group-PCA (the top 4500 weighted spatial eigenvectors) are then renormalized, eigenvalue-reweighted, and correlated to form the group average dense connectome data (91,282 × 91,282 entries). We did not perform any further preprocessing on these data beyond what had already been implemented by the HCP. Participant recruitment procedures and informed consent forms, including consent to share de-identified data, were previously approved by the Washington University Institutional Review Board as part of the HCP.

To validate the findings from the HCP dataset, we additionally analyzed structural and functional MRI data part of the Brain Genomics Superstruct Project (GSP). A comprehensive description of the GSP dataset is discussed elsewhere^53,54^. Briefly, this dataset includes 1139 participants (*M*_age_ = 21.24, SD_age_ = 2.70, 467 males, 672 females) who had undergone one structural scan (T1-weighted multi-echo MPRAGE, 1.2 mm isotropic voxels) and two 6 min functional runs at wakeful rest (gradient-echo EPI sequence, 3 mm isotropic voxels) using 3 T Siemens Tim Trio Scanners. Participants provided written informed consent in accordance with guidelines established by the Partners Health Care Institutional Review Board and the Harvard University Committee on the Use of Human Subjects in Research.

### MRI data preprocessing for the GSP dataset

Each participant’s structural data underwent intensity normalization, skull stripping, and an automated segmentation of cerebral white matter to locate the gray/white boundary via the FreeSurfer image analysis suite (v6.0), which is documented and freely available for download online (http://surfer.nmr.mgh.harvard.edu/). Defects in the surface topology were corrected^165^, and the gray/white boundary was deformed outward using an algorithm designed to obtain an explicit representation of the pial surface. Each participant’s cortical surface mesh was registered to a common spherical coordinate system^166,167^. Preprocessing of functional data was performed using the surface-based pipeline developed by Yeo and colleagues^81,168^ using a combination of FreeSurfer^169^, FSL^170^, and Advanced Normalization Tools (ANTs)^171^ routines as well as additional MATLAB functions. This pipeline consisted of the following preprocessing steps: Removal of the first four frames, slice timing correction, motion correction with rigid body translation and rotation, motion outlier detection, functional-to-structural co-registration via boundary-based registration^172^, nuisance regression, interpolation of censored frames with Lomb-Scargle periodogram^173^, and band-pass filtering [0.009, 0.08 Hz]. Volumetric data were then projected onto the FreeSurfer fsaverage6 surface space (~2 mm vertex spacing) followed by surface-constrained smoothing with a 2 mm Gaussian kernel. Subcortical voxels were resampled to the MNI152 template space (2 mm isotropic resolution) and volumetrically smoothed with a 2 mm Gaussian kernel.

We estimated framewise displacement (FD)^174^ and root-mean-square of voxel-wise differentiated signal (DVARS)^175^ using fsl_motion_outliers^176^. Volumes with FD >0.2 mm or DVARS >50 were marked as outliers (censored frames), following the criteria used by previous studies^81,168^. One frame before and two frames after these outlier volumes, as well as uncensored segments of BOLD data lasting fewer than five contiguous volumes, were also flagged as censored frames^177^. BOLD runs with more than 50% of the volumes labeled as censored frames were discarded.

To account for the effect of confounding variables, we performed linear regression separately for each BOLD run with multiple nuisance regressors, including (1) a vector of ones and linear trend, (2) six motion parameters, (3) averaged white matter signal, (4) averaged ventricular signal, along with the first-order temporal derivatives of (2), (3), and (4). The white matter mask for each participant was derived from FreeSurfer’s segmentation of their structural image, followed by three rounds of erosion before resampling to their native BOLD space. The ventricular mask was obtained similarly, but only with one round of erosion. In the event that there were fewer than 100 voxels after a round of erosion, no further erosion was performed. Regression coefficients were computed without censored frames^173^. To maintain consistency with the HCP dataset, we resampled the denoised BOLD timeseries data in the fsaverage6 space to fs_LR 32k space, after which these surface data were combined with the volumetric data to form a single whole-brain dense timeseries (.dtseries) file per run.

From the original pool of 1139 participants with two BOLD runs, we discarded 12 participants who had at least one run with more than 50% of the volumes labeled as censored frames. We additionally discarded 25 participants for whom surface resampling resulted in fewer vertices/voxels in at least one of the runs than the rest of the participants. The final GSP dataset analyzed in the current study thus consisted of 1102 individuals. For each participant, we concatenated the two BOLD runs and computed Pearson’s correlation coefficient between every pair of vertices/voxels, which was standardized via Fisher’s r-to-z transformation. Individual-level Z maps were averaged across all 1102 participants to yield the group’s average dense connectome data.

### Diffusion map embedding

We derived functional connectivity gradients of the isocortex, the cerebellum, and the hippocampus using diffusion map embedding^19,55^. Diffusion map embedding is a nonlinear data dimensionality reduction technique that enables analysis of similarity structure in functional connectivity patterns in a large number of data points (e.g., vertices/voxels) by identifying a set of low-dimensional manifolds (i.e., gradients) capturing principal dimensions of spatial variation in connectivity. Based on the group average dense connectome data in each sample, we first derived functional connectivity matrices between (1) all isocortical vertices (isocortico-isocortical symmetric matrix), (2) all cerebellar voxels and all isocortical vertices (cerebello-isocortical asymmetric matrix), and (3) all hippocampal voxels and all isocortical vertices (hippocampo-isocortical asymmetric matrix). We defined the voxels belonging to the cerebellum and the hippocampus based on a probabilistic cerebellar atlas^178^ and the Harvard-Oxford subcortical structural atlas^179,180^, respectively, with both thresholded at 50%.

We converted each functional connectivity matrix back to Pearson’s *r* values using a hyperbolic tangent function and applied row-wise thresholding to retain the top 10% connections, with all other connections set to zero. To characterize the relationship (i.e., similarity) in functional connectivity between a given pair of isocortical vertices or cerebellar/hippocampal voxels, we computed a non-negative square symmetric affinity matrix for each functional connectivity matrix. In keeping with the previous investigations, we opted to use cosine similarity to characterize the similarity structure in functional connectivity for the isocortico-isocortical and cerebello-isocortical matrices and normalized angle similarity for the hippocampo-isocortical matrices^42,181^. Finally, we used these affinity matrices as input to diffusion map embedding, which yielded ten gradients per affinity matrix identifying the dominant dimensions of spatial variation in isocortical functional connectivity as well as cerebello-isocortical and hippocampo-isocortical connectivity. In the present study, we focused on the three most dominant gradients in each structure as similar numbers of gradients have been emphasized in prior work^22,23,25,38,42^. Functional connectivity gradients were derived following the identical procedure for both the discovery and validation samples. To facilitate the comparison between samples (e.g., resolving sign indeterminacy of gradients), all connectivity gradients calculated for the validation sample were aligned to the discovery sample using Procrustes rotation (number of iterations = 10) prior to analyses.

### Statistics and reproducibility

We performed post hoc characterization of the functional gradients identified via diffusion map embedding at various levels to interpret their significance. For the hippocampal gradients, we characterized and compared the distribution of gradient values between major hippocampal subfields. To do this, we first performed automatic segmentation of hippocampal subfields on a T1-weighted structural image in the MNI152 space^182,183^. This procedure generated binary ROIs of the subiculum, CA1–3 and CA4-DG^58^, which were subsequently down-sampled to the resolution of functional data. For each of the isocortical gradients, we characterized the distribution of gradient values across the seven canonical functional networks of the isocortex^54^. Here, we adhere to the original and conventional use of these network labels and color schemes to avoid confusion, although it is important to realize that both the default mode and “limbic” networks contain agranular, limbic tissue^9,77,86^ and “limbic” network regions often appear within the default mode network^77,81^.

To interpret the cerebellar and hippocampal gradients in terms of their relations to the isocortex, we calculated functional connectivity maps between these structures and the isocortex weighted by their voxel-wise gradient values following previous studies^25,39^. For example, to characterize how a given cerebellar gradient relates to the isocortex, we computed a cerebello-isocortical connectivity map for each cerebellar voxel and multiplied it by the corresponding gradient value for that particular voxel. In this way, the pattern of functional connectivity between each cerebellar voxel and all isocortical vertices was weighted by the position of the voxel on the cerebellar gradient. These voxel-wise, gradient-weighted cerebello-isocortical connectivity values were summed over all cerebellar voxels, resulting in a single isocortical projection of the cerebellar gradient. We repeated this procedure for each gradient derived for the cerebellum and the hippocampus. To statistically assess the correspondence between these gradient-weighted functional connectivity maps of the cerebellum and the hippocampus and isocortical functional connectivity gradients, we computed vertex-wise Spearman’s rank correlations. We conducted non-parametric spin tests to derive the statistical significance of each association while controlling for spatial autocorrelations^60^. This approach follows that of prior work investigating the relationships between gradients derived from histological and functional connectivity features^7^. Importantly, this method to compare gradient-weighted functional connectivity of non-isocortical structures with isocortical functional connectivity gradients is not biased or circular because these features were not computed based on overlapping connectivity data.

We additionally performed a seed-based functional connectivity analysis to further clarify the correspondence between the isocortical, cerebellar, and hippocampal gradients. For this analysis, we first discretized each group-level functional connectivity gradient into 20 bins following prior approaches using percentile ranks of vertex-/voxel-wise gradient values^184,185^. This procedure, therefore, resulted in 20 spatially-discontiguous bins for a given gradient, each of which consisted of the same number of vertices or voxels. We computed the mean timeseries of each gradient bin, which was used to compute Pearson’s correlation coefficient for a given pair of gradients across structures within each individual participant. This analysis yielded a 20 × 20 matrix that quantifies the magnitude of functional connectivity between any given combination of gradient bins for each pair of gradients. All individual-level functional connectivity estimates were averaged to yield group-level data for each gradient pair.

For a given pair of non-isocortical (i.e., cerebellar or hippocampal) and isocortical gradients, we computed a series of Pearson’s correlation coefficients using estimates of functional connectivity calculated above. For instance, for each gradient bin generated from a non-isocortical gradient, we computed the Pearson’s correlation coefficients between isocortical gradient bin indices (bin 1–20) and functional connectivity values between the non-isocortical gradient bin and all isocortical gradient bins. Repeating this step for all non-isocortical gradient bins, we obtained 20 Pearson’s *r* values representing how functional connectivity with the 20 isocortical gradient bins changes as a function of 20 non-isocortical gradient bins. In the final step, we calculated the Pearson’s *r* value between these 20 Pearson’s *r* values indexing connectivity changes along a non-isocortical gradient and non-isocortical gradient bin indices (bin 1–20). This procedure yielded one correlation coefficient for each pair of non-isocortical and isocortical gradients quantifying how these gradients relate to each other based on functional connectivity. The gradient-weighted connectivity approach described above reveals the degree of the spatial association at a global level; the seed-based connectivity analysis described here complements that approach by quantifying the magnitude of the linear relationship between a given pair of gradients by taking into account their connectivity structure at a more precise level.

### Reporting summary

Further information on research design is available in the Nature Portfolio Reporting Summary linked to this article.

## Supplementary information

Supplementary Information

Reporting Summary

## Data Availability

The HCP dataset is publicly available at https://db.humanconnectome.org. The GSP dataset is publicly available at https://dataverse.harvard.edu/dataset.xhtml?persistentId=doi:10.7910/DVN/25833. Source data use to generate Fig. 1a and Fig. 1c are freely available online^186^.

## Code availability

All code used for data analyses in this study are freely available online^186^.
