# Supplementary material for: Correspondence of functional connectivity gradients across human isocortex, cerebellum, and hippocampus
Source: Commun Biol. 2023 Apr 12;6:401. doi: 10.1038/s42003-023-04796-0 (PMC10097701; doi:10.1038/s42003-023-04796-0)
Supplement: Supplementary file 1 — Supplementary Information [file 42003_2023_4796_MOESM1_ESM.pdf]

Supplementary Information for Katsumi et al. "Correspondence of functional connectivity gradients across human isocortex, cerebellum, and hippocampus"

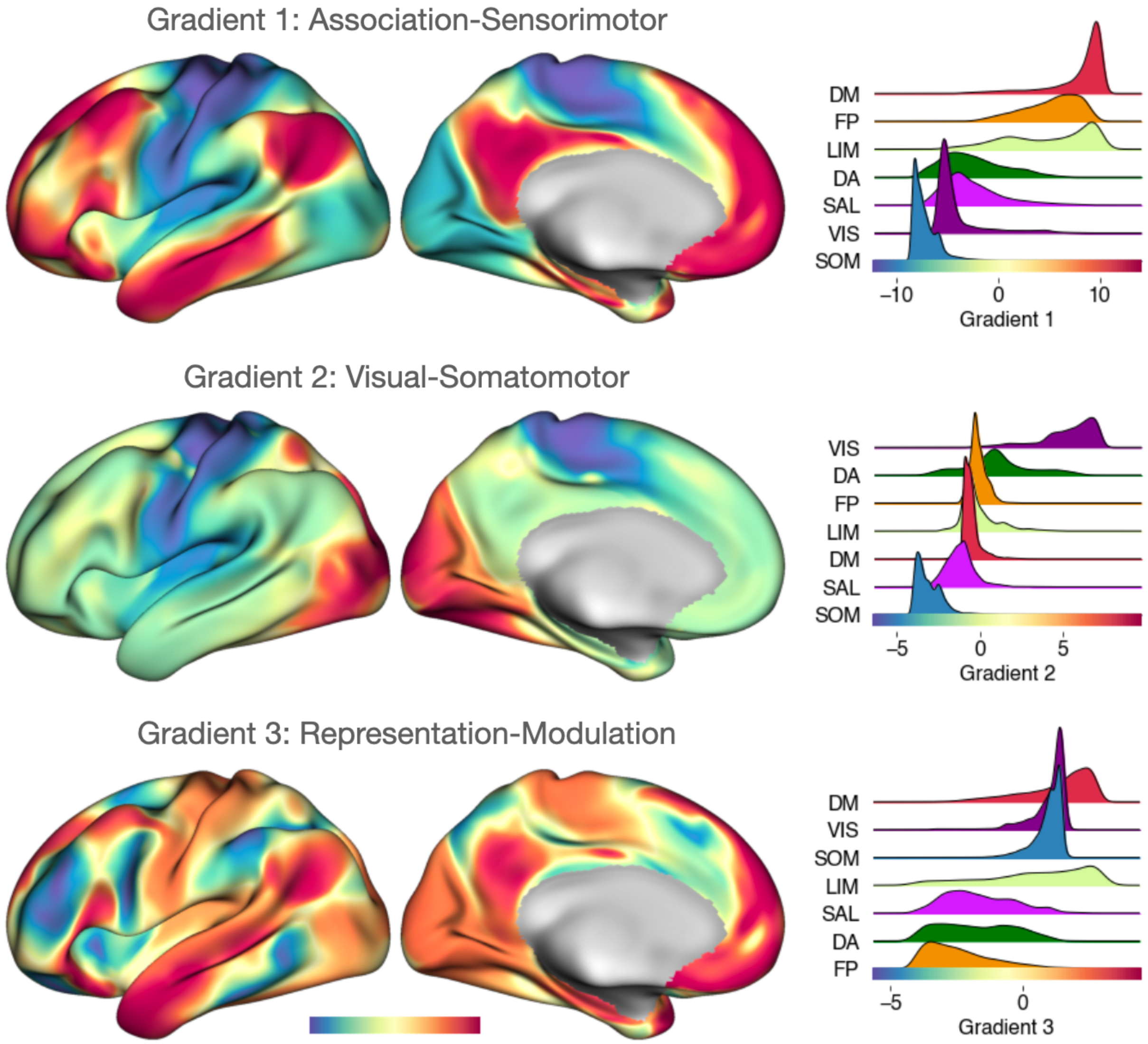

Supplementary Fig. 1. Functional connectivity gradients of human isocortex based on the primary sample (*n* = 1,003). The ridge plot depicts the distribution of gradient values based on the seven canonical functional networks of the cerebral cortex (Yeo et al., 2011). Here, we adhere to the original and conventional use of these network labels and color schemes to avoid confusion, although it is important to realize that both the default mode (DM) and "limbic" (LIM) networks contain agranular, limbic tissue (Chanes & Barrett, 2016; Kleckner et al., 2017; Paquola et al., 2021) and LIM network regions often appear within the DM network (Kleckner et al., 2017; Kong et al., 2019). DA = dorsal attention network; DM = default mode network; FP = frontoparietal network; LIM = limbic network; SAL = salience network; SOM = somatomotor network; VIS = visual network.

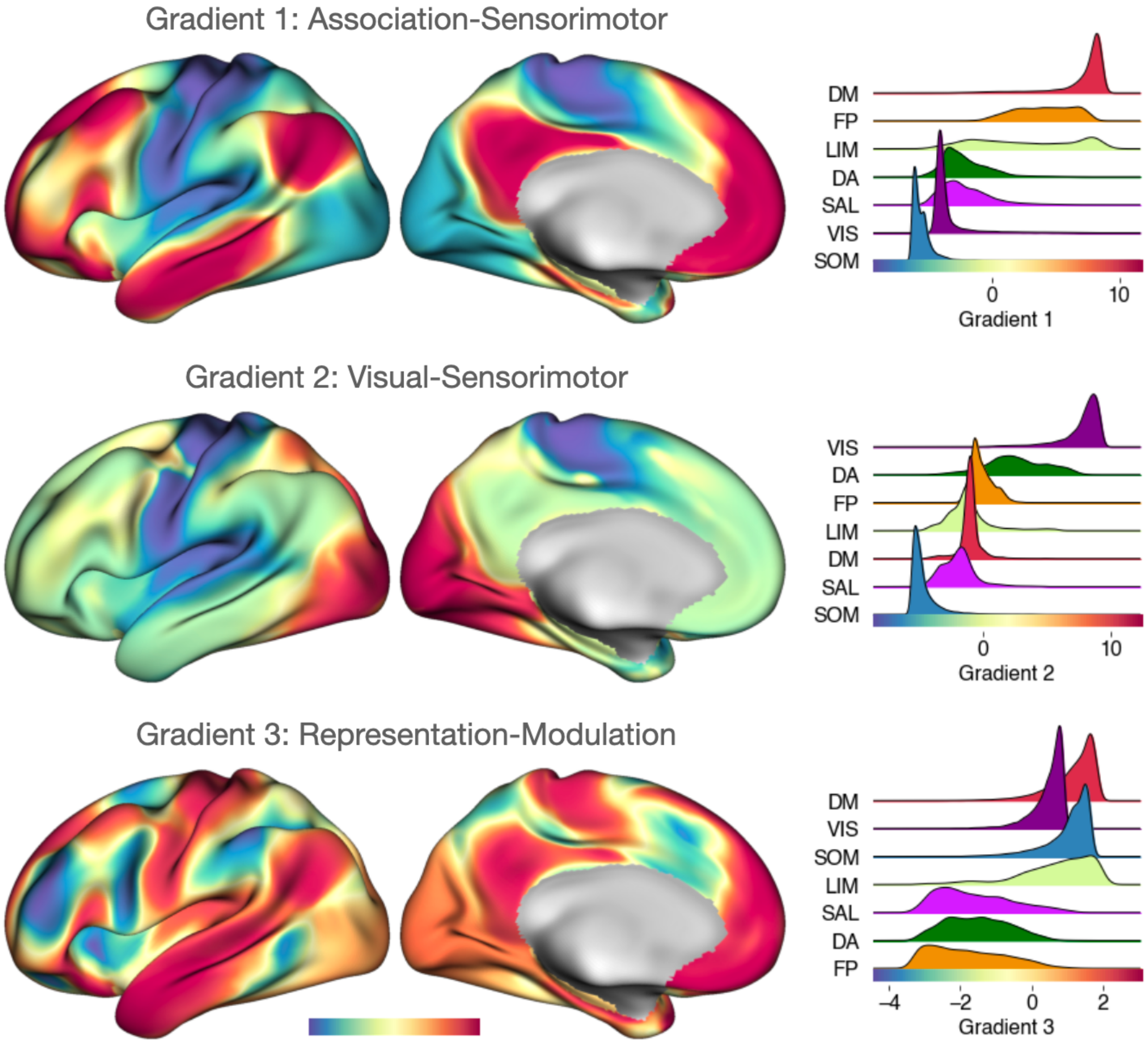

Supplementary Fig. 2. Functional connectivity gradients of human isocortex based on the validation sample (*n* = 1,102). The ridge plot depicts the distribution of gradient values based on the seven canonical functional networks of the cerebral cortex (Yeo et al., 2011). Here, we adhere to the original and conventional use of these network labels and color schemes to avoid confusion, although it is important to realize that both the default mode (DM) and "limbic" (LIM) networks contain agranular, limbic tissue (Chanes & Barrett, 2016; Kleckner et al., 2017; Paquola et al., 2021) and LIM network regions often appear within the DM network (Kleckner et al., 2017; Kong et al., 2019). DA = dorsal attention network; DM = default mode network; FP = frontoparietal network; LIM = limbic network; SAL = salience network; SOM = somatomotor network; VIS = visual network.

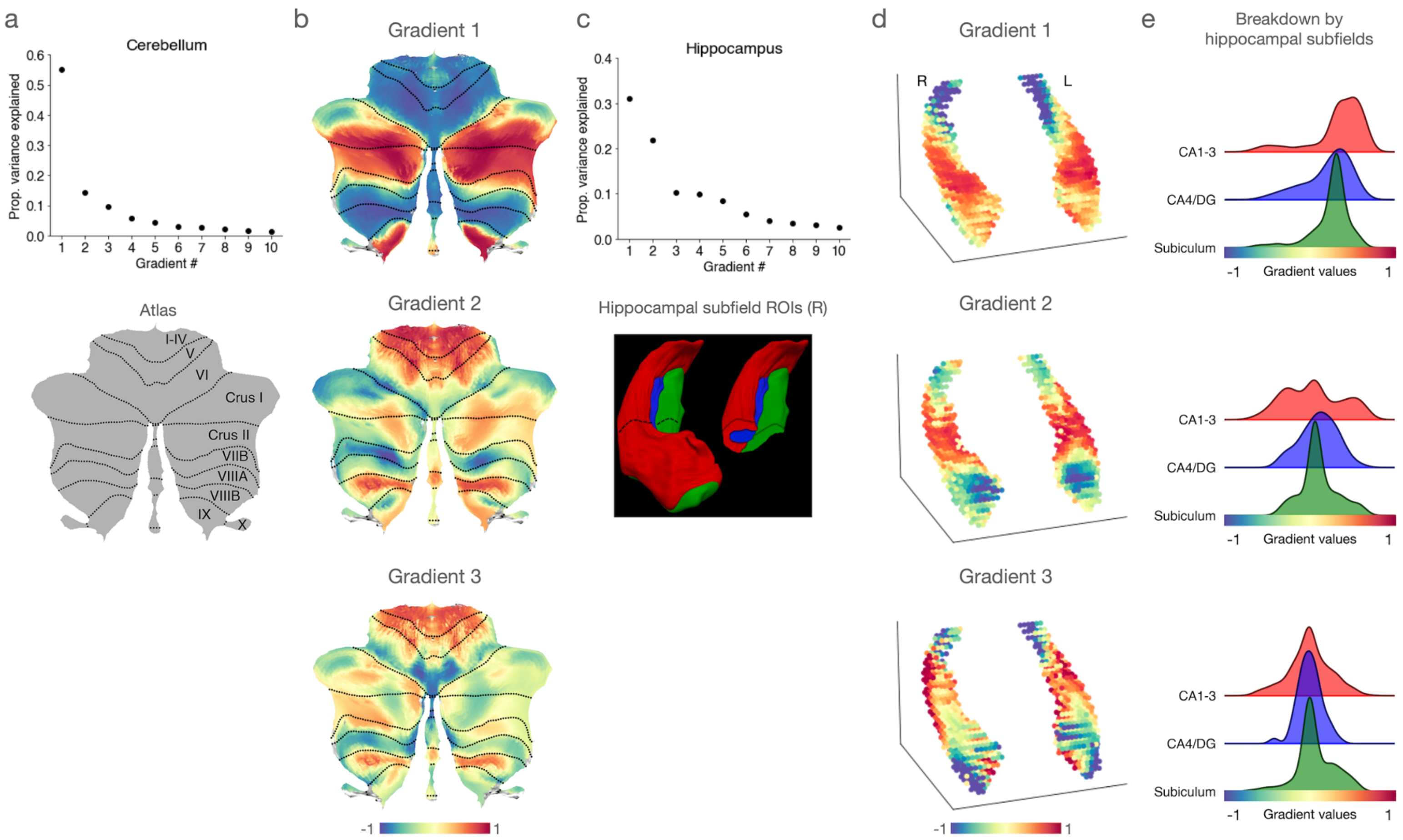

Supplementary Fig. 3. Functional connectivity gradients of human cerebellum and hippocampus based on the validation sample (*n* = 1,102). a. The scree plot illustrates the proportion of variance explained by each of the ten functional connectivity gradients derived from intrinsic connectivity between the cerebellum and the isocortex. The flatmap as a reference of the cerebellar lobules was reproduced from Guell et al. (2018) with permission. **b.** The three most dominant gradients of cerebellar-isocortical connectivity**. c.** The scree plot illustrates the proportion of variance explained by each of the ten functional connectivity gradients derived from the intrinsic connectivity between the isocortex and the hippocampus. A figure illustrating the subfields in the right hippocampus (red = CA1-3, blue = CA4-DG, green = subiculum) was reproduced from Kulaga-Yoskovitz et al. (2015) with permission. **d.** The three most dominant gradients of the hippocampal-isocortical connectivity. **e.** The ridge plot depicts the distribution of gradient values per hippocampal subfield group.

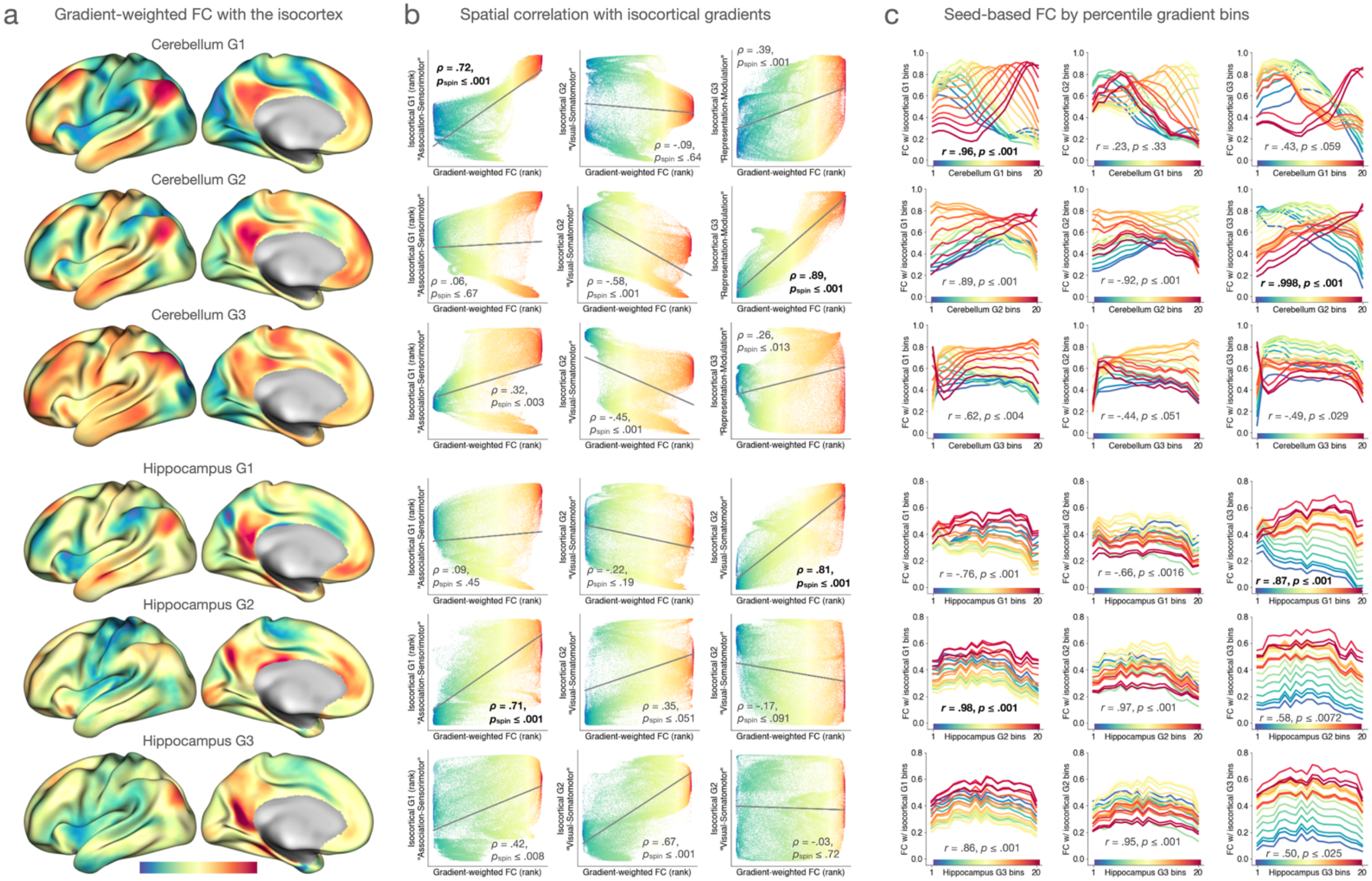

Supplementary Fig. 4. Spatial correspondence between non-isocortical and isocortical functional connectivity gradients based on the validation sample (*n* = 1,102). a. Gradient-weighted functional connectivity maps were calculated by multiplying voxel-wise isocortical functional connectivity maps of the cerebellum (or hippocampus) by cerebellar (or hippocampal) connectivity gradient values. The resulting surface maps thus characterize the topography of isocortical functional connectivity variation that each non-isocortical gradient represents. **b.** Spatial correspondence between each pair of a gradient-weighted connectivity map and an isocortical connectivity gradient was assessed by Spearman's rank correlation; *p*-values were computed while controlling for spatial autocorrelation via spin permutation tests (Alexander-Bloch et al., 2018). **c.** Seed-based analysis of functional connectivity was performed following the same procedure described in **Fig. 2C**. All *p* values are based on two-sided hypothesis testing. For each non-isocortical gradient, the strongest correlation is shown in bold font.

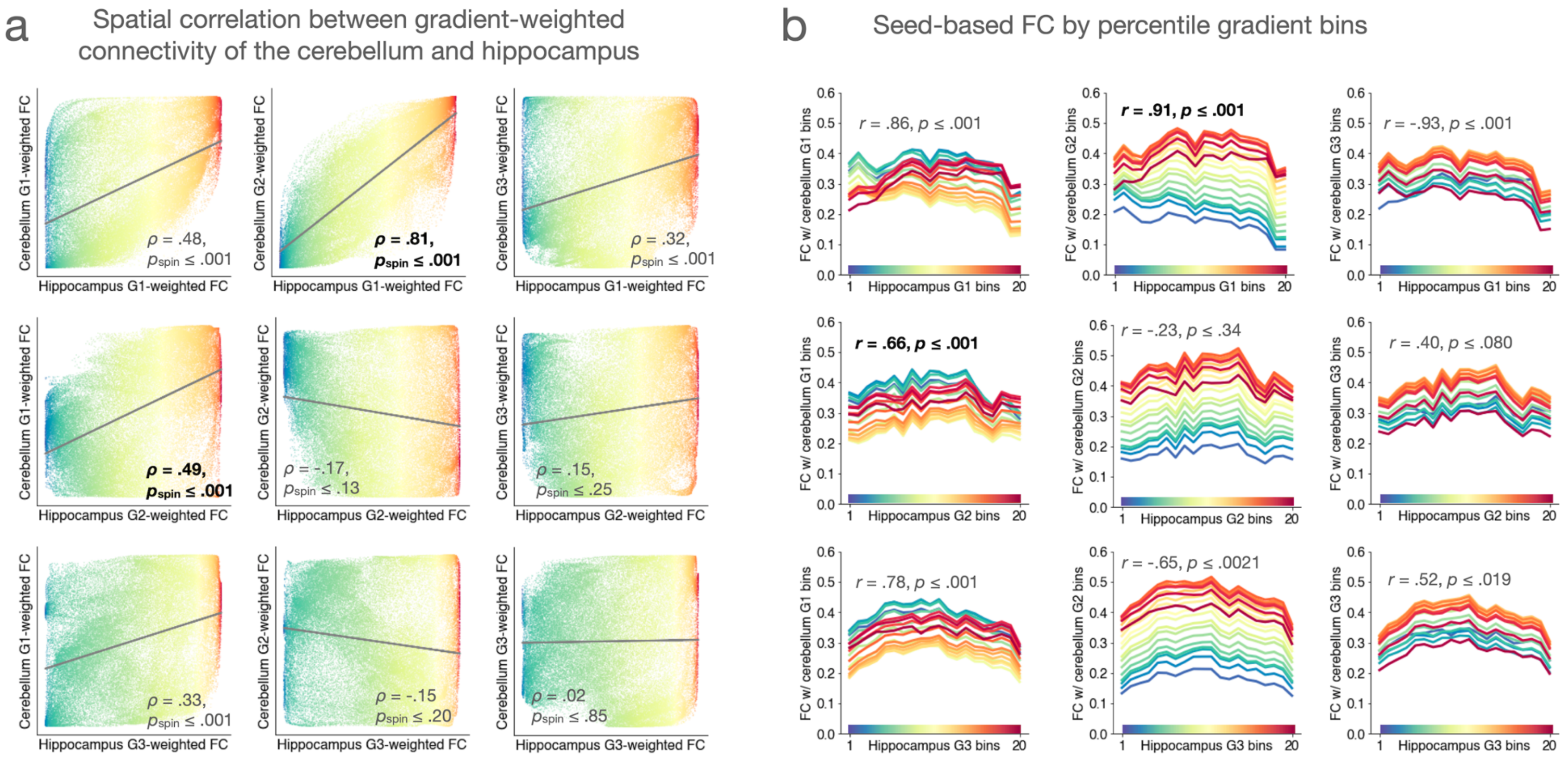

Supplementary Fig. 5. Spatial correspondence between cerebellar and hippocampal functional connectivity gradients based on the validation sample (*n* = 1,102). See **Fig. 3** in the main text for details regarding the procedure to generate these results.

Supplementary Table 1. Correspondence of functional connectivity gradients across the isocortex, cerebellum, and hippocampus at the level of single participants.

	Isocortex Gradient 1	Gradient 2	Gradient 3
Cerebellum			
Gradient 1	**99.4**	0.1	0.5
Gradient 2	2.7	0	**97.3**
Hippocampus			
Gradient 1	1.2	2.5	**96.3**
Gradient 2	**74.45**	17.86	7.68

	Cerebellum Gradient 1	Gradient 2	Gradient 3
Hippocampus			
Gradient 1	33.43	**64.27**	2.3
Gradient 2	**58.08**	13.77	28.15

Note: Each cell represents the percentage of participants showing the pattern of gradient correspondence (as measured by the strength of seed-based functional connectivity) consistent with the results of group-level analysis (denoted by **bold font**). Percentage values add up to 100% within each row.
