# Supplementary material for: Correspondence of functional connectivity gradients across human isocortex, cerebellum, and hippocampus
Source: Commun Biol. 2023 Apr 12;6:401. doi: 10.1038/s42003-023-04796-0 (PMC10097701; doi:10.1038/s42003-023-04796-0)
Supplement: Supplementary file 2 — Reporting Summary [file 42003_2023_4796_MOESM2_ESM.pdf]

nature portfolio

Corresponding author(s): Yuta Katsumi, PhD

Last updated by author(s): Mar 30, 2023

Reporting Summary

Nature Portfolio wishes to improve the reproducibility of the work that we publish. This form provides structure for consistency and transparency in reporting. For further information on Nature Portfolio policies, see our Editorial Policies and the Editorial Policy Checklist.

Statistics

For all statistical analyses, confirm that the following items are present in the figure legend, table legend, main text, or Methods section.

n/a	Confirmed	
☐	☒	The exact sample size (*n*) for each experimental group/condition, given as a discrete number and unit of measurement
☐	☒	A statement on whether measurements were taken from distinct samples or whether the same sample was measured repeatedly
☐	☒	The statistical test(s) used AND whether they are one- or two-sided<br>*Only common tests should be described solely by name; describe more complex techniques in the Methods section.*
☐	☒	A description of all covariates tested
☐	☒	A description of any assumptions or corrections, such as tests of normality and adjustment for multiple comparisons
☐	☒	A full description of the statistical parameters including central tendency (e.g. means) or other basic estimates (e.g. regression coefficient) AND variation (e.g. standard deviation) or associated estimates of uncertainty (e.g. confidence intervals)
☐	☒	For null hypothesis testing, the test statistic (e.g. *F*, *t*, *r*) with confidence intervals, effect sizes, degrees of freedom and *P* value noted<br>*Give P values as exact values whenever suitable.*
☒	☐	For Bayesian analysis, information on the choice of priors and Markov chain Monte Carlo settings
☒	☐	For hierarchical and complex designs, identification of the appropriate level for tests and full reporting of outcomes
☐	☒	Estimates of effect sizes (e.g. Cohen's *d*, Pearson's *r*), indicating how they were calculated

Our web collection on statistics for biologists contains articles on many of the points above.

Software and code

Policy information about availability of computer code

Data collection	All MRI data used to generate the results of this study were collected using 3 Tesla MRI scanners with specific sequences/scanning parameters. For more information, please refer to the relevant publications by the Human Connectome Project (doi: 10.1016/j.neuroimage.2013.05.041) and the Brain Genomics Superstruct Project (doi: 10.1038/sdata.2015.31) teams.
Data analysis	Preprocessing of MRI data was performed using custom code written by the CBIG Group, which is freely available online (https://github.com/ThomasYeoLab/CBIG/tree/master/stable_projects/preprocessing/CBIG_fMRI_Preproc2016). Diffusion map embedding on functional MRI data was performed using custom code freely available online (https://github.com/satra/mapalign).

For manuscripts utilizing custom algorithms or software that are central to the research but not yet described in published literature, software must be made available to editors and reviewers. We strongly encourage code deposition in a community repository (e.g. GitHub). See the Nature Portfolio guidelines for submitting code & software for further information.

Data

Policy information about availability of data

All manuscripts must include a data availability statement. This statement should provide the following information, where applicable:

- Accession codes, unique identifiers, or web links for publicly available datasets
- A description of any restrictions on data availability
- For clinical datasets or third party data, please ensure that the statement adheres to our policy

All MRI data used to generate the results of this work are freely available from the Human Connectome Project (https://db.humanconnectome.org/) and the Brain Genomics Superstruct Project (https://dataverse.harvard.edu/dataverse/GSP).

Field-specific reporting

Please select the one below that is the best fit for your research. If you are not sure, read the appropriate sections before making your selection.

☒ Life sciences ☐ Behavioural & social sciences ☐ Ecological, evolutionary & environmental sciences

For a reference copy of the document with all sections, see nature.com/documents/nr-reporting-summary-flat.pdf

Life sciences study design

All studies must disclose on these points even when the disclosure is negative.

Sample size	We processed and analyzed all available data provided by the HCP and the GSP; no sample size calculation was performed specifically for the current study, consistent with previous studies utilizing these large-scale datasets.
Data exclusions	From the original pool of 1,139 participants with two BOLD runs in the GSP dataset, we excluded 12 participants who had at least one run with more than 50% of the volumes labeled as censored frames. We additionally excluded 25 participants for whom surface resampling resulted in fewer vertices/voxels in at least one of the runs than the rest of the participants. The final GSP dataset analyzed in the current study thus consisted of 1,102 individuals.
Replication	We performed all analyses in two large, independent datasets with n=1000+ to ensure replicability of the reported results. We provided a possible explanation for results that were not consistently identified across two samples (see Discussion).
Randomization	No experimental groups were constructed specifically in the current study.
Blinding	No blinding was performed as the current study did not involve experimental manipulations.

Reporting for specific materials, systems and methods

We require information from authors about some types of materials, experimental systems and methods used in many studies. Here, indicate whether each material, system or method listed is relevant to your study. If you are not sure if a list item applies to your research, read the appropriate section before selecting a response.

Materials & experimental systems

n/a	Involved in the study	
☒	☐	Antibodies
☒	☐	Eukaryotic cell lines
☒	☐	Palaeontology and archaeology
☒	☐	Animals and other organisms
☒	☐	Human research participants
☒	☐	Clinical data
☒	☐	Dual use research of concern

Methods

n/a	Involved in the study	
☒	☐	ChIP-seq
☒	☐	Flow cytometry
☐	☒	MRI-based neuroimaging

Magnetic resonance imaging

Experimental design

Design type	Resting-state functional MRI
Design specifications	Four runs of a 15 min scan (HCP); two runs of a 6 min scan (GSP).
Behavioral performance measures	No behavioral measures were collected during resting-state functional MRI scan sessions.

Acquisition

Imaging type(s)	Structural, functional
Field strength	3 Tesla
Sequence & imaging parameters	HCP structural: 3D MPRAGE, TR=2400ms, TE=2.14ms, TI=1000ms, FA=8 degrees, FoV=224*224, voxel size=0.7mm isotropic; HCP functional: gradient-echo EPI, TR=720ms, TE=33.1ms, FA=52 degrees, FoV=208*180, matrix size=104*90, slice thickness=2mm, multiband factor=8; GSP structural: 3D MEMPRAGE, TR=2200ms, TE=1.5/3.4/5.2/7.0ms, TI=1100ms, FA=7 degrees, voxel size=1.2mm isotropic; GSP functional: gradient-echo EPI, TR=3000ms, TE=30ms, FA=85

degrees, slice thickness=3mm.

Area of acquisition: Whole-brain

Diffusion MRI: ☐ Used ☒ Not used

Preprocessing

Preprocessing software

HCP: We used the preprocessed data made available as part of the HCP1200 2017 data release; no additional preprocessing was performed. GSP: We used custom preprocessing code developed by the CBIG group, which is freely available online (https://github.com/ThomasYeoLab/CBIG/tree/master/stable_projects/preprocessing/CBIG_fMRI_Preproc2016). Specifically, this pipeline included the following steps: Removal of the first four frames, slice timing correction, motion correction with rigid body translation and rotation, motion outlier detection, functional-to-structural co-registration via boundary-based registration, nuisance regression, interpolation of censored frames with Lomb-Scargle periodogram, and band-pass filtering [0.009, 0.08 Hz]. Volumetric data were projected onto the FreeSurfer fsaverage6 surface space (~2 mm vertex spacing) followed by surface-constrained smoothing with a 2 mm Gaussian kernel. Subcortical voxels were resampled to the MNI152 template space (2 mm isotropic resolution) and volumetrically smoothed with a 2 mm Gaussian kernel.

Normalization

Surface-space data were spatially normalized to the fsaverage6 template (then subsequently to fs_LR 32k space); volumetric data were spatially normalized to the MNI152 6th gen template. All correlation coefficients were standardized via Fisher's r-to-z transformation.

Normalization template

fsaverage6, fs_LR 32k, MNI152 6th Gen.

Noise and artifact removal

To account for the effect of confounding variables, we performed linear regression separately for each BOLD run with multiple nuisance regressors, including (1) a vector of ones and linear trend, (2) six motion parameters, (3) averaged white matter signal, (4) averaged ventricular signal, along with the first-order temporal derivatives of (2), (3), and (4). The white matter mask for each participant was derived from FreeSurfer's segmentation of their structural image, followed by three rounds of erosion before resampling to their native BOLD space. The ventricular mask was obtained similarly, but only with one round of erosion. In the event that there were fewer than 100 voxels after a round of erosion, no further erosion was performed. Regression coefficients were computed without censored frames.

Volume censoring

Frames to be censored were defined by any frame exhibiting framewise displacement (FD) > 0.2 or root-mean-square of voxel-wise differentiated signal (DVARS) > 50. One frame before and two frames after these outlier volumes, as well as uncensored segments of BOLD data lasting fewer than five contiguous volumes, were also flagged as censored frames. BOLD runs with more than 50% of the volumes labeled as censored frames were discarded.

Statistical modeling & inference

Model type and settings

Univariate, spatial permutation test controlling for spatial autocorrelation

Effect(s) tested

Hippocampal subfields, functional connectivity gradients

Specify type of analysis: ☐ Whole brain ☐ ROI-based ☒ Both

Anatomical location(s): Cerebral cortex (isocortex, hippocampus) and cerebellum

Statistic type for inference (See Eklund et al. 2016)

No voxel-wise or cluster-wise inference was performed.

Correction

No correction for multiple comparisons was implemented for the analysis of gradient value distributions across hippocampal subfields and spatial correlation of gradient values/gradient-weighted connectivity values. Correction for spatial autocorrelation was implemented via permutation testing (spin test).

Models & analysis

n/a	Involved in the study	
☐	☒	Functional and/or effective connectivity
☒	☐	Graph analysis
☒	☐	Multivariate modeling or predictive analysis

Functional and/or effective connectivity

Functional connectivity was estimated by calculating the Pearson correlation coefficient.
